# Total Ginsenosides of Radix Ginseng Modulates Tricarboxylic Acid Cycle Protein Expression to Enhance Cardiac Energy Metabolism in Ischemic Rat Heart Tissues 

**DOI:** 10.3390/molecules171112746

**Published:** 2012-10-29

**Authors:** Jing-Rong Wang, Hua Zhou, Xiao-Qin Yi, Zhi-Hong Jiang, Liang Liu

**Affiliations:** 1 State Key Laboratory of Quality Research in Chinese Medicine, Macau Institute for Applied Research in Medicine and Health, Macau University of Science and Technology, Macau, China; Email: jrwang@must.edu.mo (J.-R.W.); hzhou@must.edu.mo (H.Z.); zhjiang@must.edu.mo (Z.-H.J.); 2 School of Chinese Medicine, Hong Kong Baptist University, Hong Kong, China; Email: xqyi@hkbu.edu.hk

**Keywords:** total ginsenosides, proteomics, cardio-protection, energy metabolism

## Abstract

To elucidate the underlying mechanism of cardio-protective activity of the total ginsenosides (TGS) of Radix Ginseng, proteomic analysis using two-dimensional gel electrophoresis (2-DE) and MALDI-TOF-TOF-MS techniques was employed for identifying the underlying targets of TGS on improvement of the energy metabolism of isolated rat heart tissues perfused in Langendorff system under ischemia-reperfusion injury conditions. The image analysis results revealed 11 differentially expressed proteins in the TGS-treated heart tissues; these proteins, including LDHB and ODP-2, were found to be closely related to the function of tricarboxylic acid (TCA) cycle that plays pivotal roles in cardiac energy metabolism. It is thus concluded that improvement of cardiac energy metabolism via activating proteins in TCA cycle could be the major action pathway and targets of TGS activity against rat heart tissue injury.

## 1. Introduction

Ischemic heart diseases are among the leading causes of death in humans in the industrialized countries. Although ischemic injury of heart tissues can be greatly recovered by rapid reperfusion, severe side effects such as cardiac over-contractile function, arrhythmia, endothelial dysfunction, and myocardial infarction often occur due to reperfusion [1]. Moreover, endothelial dysfunction in ischemic heart tissues may lead to loss of the endothelium-derived dilator of nitric oxide (NO), decrease of blood perfusion in the tissues, myocytes apoptosis, non-infectious inflammation and other complicated cardiac pathological status [2]. Therefore, ischemia/reperfusion (I/R) injury has been evident as one of the most pivotal pathological factors of human ischemic heart diseases. Also, both *in vivo* and *in vitro* I/R models are often employed in laboratory for examining pathogenesis or therapeutics of human ischemic heart conditions [3]. 

Ginsenosides have been demonstrated as the major chemical components responsible for the most observed bioactivities of ginseng herb in clinical usage [4]. *In vivo* and *in vitro* investigations have revealed a number of significant actions of ginsenosides and ginseng extracts in cardio-protection, such as reducing myocardial ischemia-reperfusion induced damage via NO pathway in rats and mice [5], slowing down deterioration of cardiac contractions, preventing development of arrhythmias [6] and relaxing the muscles of the aorta [7]. Our previous studies showed that total ginsenosides (TGS) significantly increased coronary artery flow both in the basal perfusion and I/R injury condition of the isolated rat hearts in Langendorff system through activating Akt-eNOS signaling, which suggests that TGS could benefit patients with ischemic heart conditions [8]. To uncover the molecular mechanisms underlying the myocardial protection of TGS, proteomic analysis with two-dimensional gel electrophoresis (2-DE) and MALDI-TOF-TOF-MS techniques was performed on isolated rat heart tissues perfused in Langendorff system under ischemia-reperfusion injury conditions with or without TGS treatment in the current study. 

## 2. Result and Discussion

To optimize the protein extraction method, especially the membrane proteins that are usually difficult to dissolve due to their many hydrophobic residues, different extraction buffers (Tris, urea and thiourea) with various detergents (SDS, CHAPS, ASB-14) and different concentrations of the chaotropic agents were examined at the beginning of the analysis. The extraction efficacy was evaluated by 1D-SDS PAGE and 2-DE, showing that 50 mM Tris with 2% SDS could achieve the maximum extraction efficacy (data not shown).

**Figure 1:**
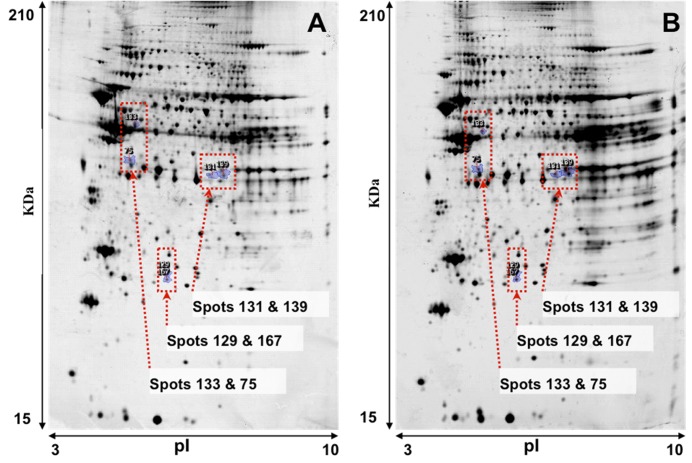
Representative images of 2-DE gel presenting the proteome of the left ventricle tissues of rats in the control (**A**) and TGS-treated group (**B**).

**Figure 2:**
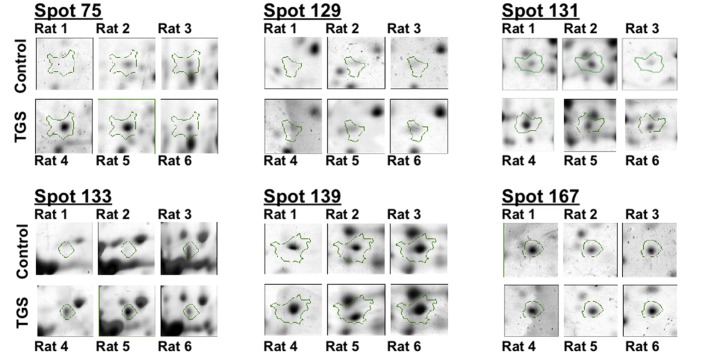
Differential expressions of six identified proteins by 2-DE analysis. Enlarged 2-DE gel images of the individual left ventricle tissues from three hearts either in the control group and or in the TGS-treated group.

**Figure 3:**
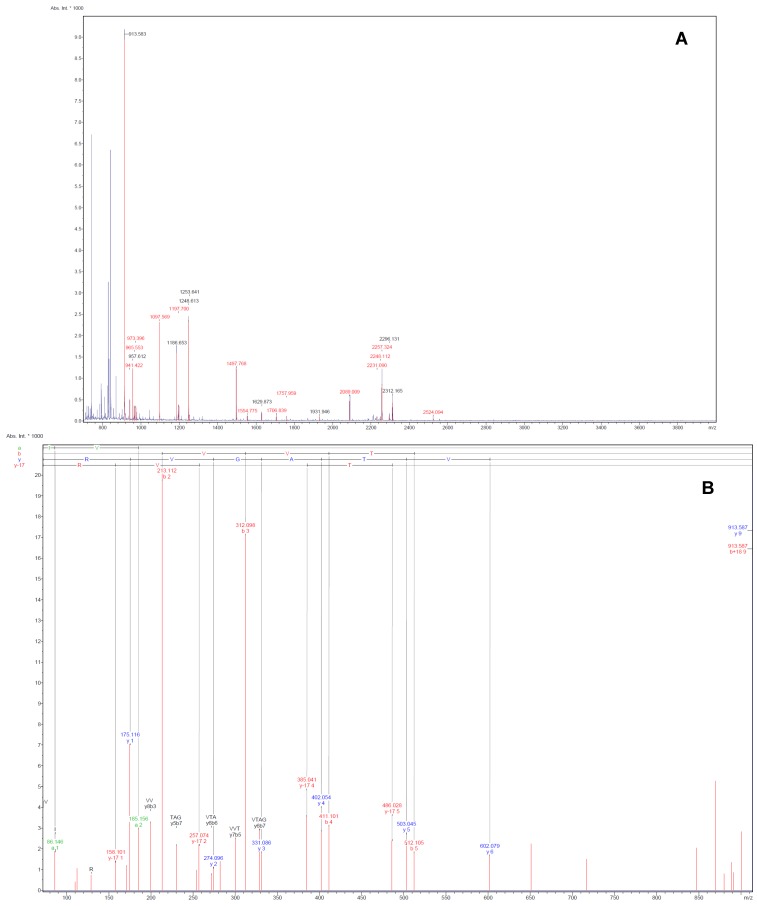
Illustrative examples of Peptide Mass Fingerprint (PMF, **A**) of spot 75 and the MS/MS spectrum (**B**) of the mono-isotopic ion of *m/z* 913.6 The MS/MS spectrum shows the *m/z* peaks of the b- and y-ions, which denotes fragmentations at the amide with charged retention on the N or C-terminus of the tryptic peptide.

Representative images of 2-DE gel presenting the proteomes of the left ventricle tissues of isolated rat hearts and TGS-treated hearts are shown in Figure 1. Image analysis with Progenesis SameSpots revealed that typically 1,000 protein spots were detected per gel and a set of 12 gel images (three biological replicates for each group and two analytical replicates for each sample, *i.e.*, three ventricles from the control group and three ventricles from TGS group, gels were run in duplicate for each ventricles) were produced. Using a *p*-value of <0.05 as cut-off with one-way ANOVA analysis, 11 differentially expressed protein spots were found in the TGS-treated group, and six proteins as shown in Figure 2 were identified successfully by MALDI-TOF-MS. The identification of the proteins was achieved by using both Peptide Mass Fingerprint (PMF) approach and MS/MS analysis of the matched peptide (Figure 3). For the five remaining spots, no adequate peptide mapping could be performed either due to too low amount of proteins or due to technical limitations. A list of the identified proteins together with their accuracy of identification as indicated by the Mowse score and the volume ratios between the control and TGS-treated groups are given in Table 1. 

**Table 1:** Identification of the differentially expressed proteins in rat left ventricle tissues induced by TGS treatment.

No.	Swiss-Prot accession number	Protein names		Functions	Folds of change ^a^	*P* value *^b^*	Theoretical		Sequence Coverage (%) *^c^*	Mowse Score
							*Mr*	*pI*		
75	P42123	LDHB-RAT	L-lactate dehydrogenase B chain	Glucose metabolism	2.1	0.035	36.8	5.70	30	303
129	0BB767	PARK7-RAT	Protein DJ-1	Anti-oxidative stress	1.7	0.016	20.2	6.32	25	155
131	035077	GPDA-RAT	Glycerol-3-phosphate dehydrogenase [NAD+], cytoplasmic	Mitochondrial respiratory chain	1.7	0.038	38.1	6.16	17	137
133	Q3B7V7	ODP2-RAT	Dihydrolipoyllysine-residue acetyltransferase component of pyruvate dehydrogenase complex	TCA cycle	1.7	0.049	67.6	8.76	9	220
139	P07943	ALDR-RAT	Aldose reductase	Glucose metabolism	1.6	0.039	36.2	6.26	37	365
167	P36972	APT-RAT	Adenine phosphoribosyltransferase	Nucleotide Metabolism	1.5	0.002	19.7	6.17	31	244

*^a^* Denoted as averaged V_TGS_/V_model _where V_TGS_ and V_model_ represent normalized spot volume in TGS-treated group and model control group, respectively; *^b^* Compared with one-way ANOVA analysis; *^c^* Percentage of the protein sequence covered by the matching peptides identified.

As seen in Table 1, proteins that were up-regulated in TGS-treated ventricles are at the most responsible for glucose metabolism, citric acid cycle, mitochondrial respiratory chain, and anti-oxidant proteins, among which most of them are associated with ATP production, indicating that a metabolic regulation of cardiac energy could be induced by TGS treatment. Such metabolic regulation by TGS seems to be especially observed at pyruvate level as suggested by the up-regulation of LDHB (spot 75) and ODP-2 (alternative name, PDC-E2).

**Figure 4:**
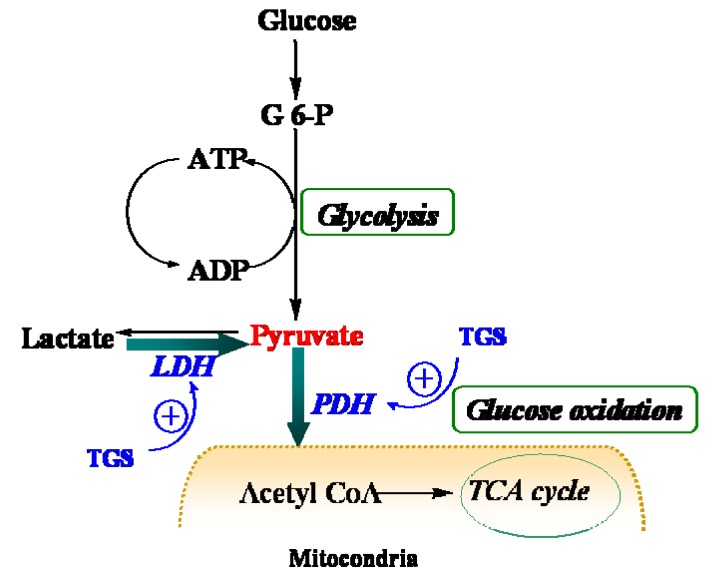
Schematic depiction of myocardial metabolism together with modulation by TGS treatment. G 6-P, Glucose 6-phosphate; LDH (lactate dehydrogenase); PDH, pyruvate dehydrogenase; TCA, tricarboxylic acid.

LDHB, a subunit of LDH, has long been recognized to favor the reaction of lactate to pyruvate [9] although it can also catalyze the reverse reaction too. In the present study, it was found that dihydrolipoyllysine-residue acetyltransferase component of PDH complex (PDC-E2) was up-regulated by TGS treatment, which may benefit pyruvate conversion. Acceleration of pyruvate metabolism was expected to shift the equation of lactate and pyruvate towards acetyl coenzyme A (Figure 4). The pronounced effect of TGS on expression of LDHB and PDC-E2 showed a signal of increased glucose oxidation as energy generation for myocardium. During ischemia, the increased glucose oxidation might have therapeutic benefits, *i.e.*, a greater ATP yield for given oxygen consumption compared to fatty acid, as well as a greater rate of pyruvate oxidation and less lactate accumulation [10]. These results have collectively suggested an overall improvement on the cardiac energy metabolism of TGS through modulation of TCA in the isolated rat hearts. TCA cycle is a series of enzyme-catalyzed chemical reactions that form a key part of aerobic respiration in cells. This cycle is also called the Krebs cycle and the citric acid cycle. The greatly simplified cycle starts with pyruvate, which is the end product of glycolysis, the first step of all types of cell respiration. Therefore, this might be one of the major molecular mechanisms of the TGS’s actions against heart tissues injury caused by ischemia-reperfusion. Furthermore, ginseng has been using widely as a general restorative for the “qi” or vital energy in the body in traditional Chinese medicine (TCM). Such restoration potency might be partially attributable to the energy metabolism improvement of ginsenosides. Thus, our current study has also provided certain evidences of mechanisms of ginseng for its traditional restorative function of “qi”. 

In addition, we previously found that increased activation of PI3K/Akt/eNOS signaling pathways was accompanied with increased coronary artery flow and decreased myocardial ischemia reperfusion injury when TGS or ginseng extract were applied to isolated hearts of rats [5,8]. Recent studies have demonstrated that activation of the PI3K/Akt signaling pathway not only protected the heart from ischemia reperfusion injury through eNOS dependent mechanisms, but also bestowed a bioenergetic advantage to the cells by up-regulating mitochondrial respiratory capacity through the 4E-BP1-mediated protein translation pathway [11]. In addition, PI3K/Akt signaling up-regulates the expression of the glucose transporter GLUT1 and increases glucose intake in T cells [12,13]. Therefore, our current finding that TGS treatment results improvement of cardiac energy metabolism via activating proteins in TCA cycle could be closely related with the activation of PI3K/Akt/eNOS pathways induced by TGS. However, the exact role of PI3K/Akt/eNOS in the regulation of energy metabolism caused by TGS remains an intriguing issue and needs further investigation.

In addition, the proteomic data revealed up-regulation of aldose reductase (ALDR) in TGS-treated hearts. ALDR is a multi-functional enzyme that catalyzes reduction of a wide range of endogenous and xenobiotic aldehydes and their glutathione conjugates with high efficiency. Also, ALDR may prevent endoplasmic reticulum stress induced by excessive accumulation of aldehyde-modified proteins in the ischemic heart, hence to protect heart tissues against ischemic injury [14]. However, ALDR was also reported to be up-regulated in inflammatory response, and inhibition of aldose reductase shows potential anti-inflammation and cell protective effects. Furthermore, overexpression of ALDR in cardiomyocytes leads to cardiac dysfunction, suggesting that pharmacological inhibition of ALDR will be beneficial during ischemia and in some forms of heart failure [15]. Since the role of ALDR in ischemia reperfusion injury is controversial, more evidence is needed to determine the exact role of up-regulated ALDR accompanying TGS treatment in ischemia-reperfused heart.

## 3. Experimental

### 3.1. Preparation of Total Ginsenosides (TGS)

TGS was prepared from Asian ginseng (AG), *Panax ginseng* C.A. Meyer and quantified in our laboratory as described previously [8]. Briefly, TGS was prepared from AG roots by using refluxing ethanol extraction and column chromatography over D101 polymeric resin. HPLC analysis on TGS was performed and twelve ginsenosides, namely, Rg_1_, Re, R_0_, malonyl Rb_1_ (mRb_1_), malonyl Rc (mRc), malonyl Rb_2_ (mRb_2_), malonyl Rd (mRd), Rf, Rb_1_, Rc, Rb_2_ and Rd, were identified and quantitatively determined, corresponding to nearly 70% of the constituents of TGS. 

### 3.2. Animals and Chemical Reagents

Male Sprague-Dawley (SD) rats of 200–220 g were purchased from the Laboratory Animal Services Center of the Chinese University of Hong Kong (Hong Kong, China). All animals were acclimated in the laboratory for ≥1 week under 12 h light and 12 h dark cycle at room temperature of 22 ± 1 °C prior to the experiment. Animals were housed four per cage with food and water provided *ad libitum.* Animal care and treatment procedures conformed to the Institutional Guidelines and Animal Ordinance of the Department of Health of the Hong Kong Special Administration Region. 

Urea, thiourea, dithiothreitol (DTT), iodoacetamide, Tris, PlusOne Glycerol (87%), PlusOne Glycine, sodium dodecylsulfate (SDS) were purchased from GE Healthcare Bio-Sciences (Björkgatan, Uppsala, Sweden). 

### 3.3. Rat Heart Ischemia-reperfusion and TGS Treatment in Langendorff System

Rat hearts were perfused in Langendorff system as described previously [8]. In brief, the rat hearts were isolated and retrogradely perfused with Krebs-Henseleit (K-H) buffer equilibrated with 95% O_2_ and 5% CO_2_ (pH 7.4) in a Langendorff perfusion system (AD Instruments, Sydney, Australia) at a constant coronary perfusion pressure of 65 ± 1 mmHg at 37 °C for 20–30 min (stabilization), then perfused at 20 ± 1 mmHg for 40 min to produce a global low flow ischemia, and finally reperfused at 65 ± 1 mmHg for 10 min. The hearts were randomly perfused with K–H buffer, TGS in K–H buffer at 50 mg/L for 60 min, starting from the last 10 min of stabilization until the end of reperfusion. The ischemia and reperfusion as well as the cardioprotection of TGS treatment were confirmed by change of coronary perfusion flow rate as shown in our previous reports [8]. At the end of reperfusion, the hearts were collected and the left ventricles were quickly separated, weighed, frozen in liquid nitrogen, and stored at −80 °C for protein extraction. 

### 3.4. Protein Extraction

Small pieces (200–300 mg) of left ventricle from the ischemia-reperfused rat heart treated-with or without TGS were homogenized in 2 mL of sample buffer (50 mM Tris-HCl (pH 6.8), 2% (w/v) SDS, 10% (w/v) glycerol, 100 mM DTT and protease inhibitor mix (GE Healthcare) by using the grinding kit (GE Healthcare) at 4 °C. The homogenates were centrifuged for 1 h (2,500 × g) at 4 °C. The resulting supernatants (total proteins) were stored at −80 °C for further analysis. Prior to 2-DE analysis, the proteins were selectively precipitated using PlusOne 2D-Clean Up Kit (GE Healthcare, USA) to remove non-protein impurities and re-dissolved in lysis buffer (7 M urea, 2 M thiourea, 4% CHAPS). Protein concentration was determined by Plus One 2D-Quant kit (GE Healthcare). 

### 3.5. Two-Dimensional Gel Electrophoresis (2-DE)

For sample loading, proteins (100 μg) were added into the rehydration buffer [(7 M urea, 2 M thiourea, 4% (w/v) CHAPS, 0.2% (v/v) pharmalyte 3–10, 1.2% (v/v) destreak reagent (GE Healthcare)) to a final volume of 250 μL. pH 3–10 nonlinear (NL) IPGstrips (13 cm) were rehydrated with the sample in the standard strip holder (GE Healthcare) under voltage of 30 V for 10 h. The first dimension was run at 50 μA/strip on the Ettan IPGphor 3 System (GE Healthcare) with following the voltage increase protocol: 250 V for 1 h, linear gradient to 500 V in 1 h, stepwise to 8,000 V in 1 h and 8,000 V until 64 KVh was reached. After the first dimension isoelectrifocusing, strips were equilibrated in SDS equilibration buffer (6 M urea, 75 mM Tris-HCl (pH 8.8), 29.3% (v/v) glycerol, 2% (w/v) SDS, 0.002% (w/v) bromophenol blue) containing 10 mg/mL DTT for 15 min, and thereafter in SDS equilibration buffer containing 25 mg/mL iodoacetamide for 15 min. After equilibration, proteins were separated on 12% polyacrylamide gel in SE 600 standard vertical electrophoresis unit (Amersham Biosciences, Björkgatan, Uppsala, Sweden). The IPG strips were sealed in place with 1% agarose solution. The second dimension electrophoresis was performed at 10 V/gel for the initial migration step and 25 V/gel for the separation step at room temperature until the bromophenol blue front reached the bottom of the glass plate (total duration 4–6 h). The temperature was kept at 22 °C.

After 2-DE separation, the gels were fixed for at least 30 min in 40% ethanol and 10% acetic acid. Then the gels were stained by using the silver staining kit (GE Healthcare) according to the protocol that is compatible with subsequent protein identification. The silver stained gels were scanned with GE Image Scanner III and analyzed with the Progenesis SameSpots software (Nonlinear Dynamics Ltd, Newcastle upon Tyne, UK). 

### 3.6. In-Gel Trypsin Digestion

Protein spots showing significantly altered expression levels between the two groups of samples were excised in duplicate from the silver-stained gels and identified by MALDI-TOF-MS/MS (Bruker Autoflex III MALDI TOF-TOF MS). Briefly, the gel plugs were destained using a destaining solution (1:1, 30 mM potassium ferricyanate: 100 mM sodium thiosulfate, freshly mixed before use) until silver stain fade. After dehydration with acetonitrile, which was by then removed, the gel plugs were dried in vacuum centrifuge. Then, 0.5 mL of 10 mM DTT in 10 mM NH_4_HCO_3_ was added and the proteins were reduced for 15 min at 56 °C. The DTT solution was subsequently replaced with 0.5 mL 55 mM iodoacetamide in 10 mM NH_4_HCO_3_. After 20 min incubation at room temperature in the dark, the gel plugs were washed with 10 mM NH_4_HCO_3_ and dehydrated with acetonitrile followed by drying in vacuum centrifuge. The completely dried gel plugs were incubated with trypsin (trypsin gold, mass spectrometry grade, Promega, Madison, WI, USA) solution (5 μg/mL in 10 mM NH_4_HCO_3_) overnight at 37 °C. Peptides were successively extracted with 1% TFA in 50% acetonitrile and 1% TFA in 100% acetonitrile.

### 3.7. Mass Spectrometry and Database Searching

MALDI samples were prepared by spotting 1–2 μL digested solution onto a thin layer of α-cyano-4-hydroxy-cinnamic acid on 600 μm AnchorChip MALDI probe (Bruker Daltonics). After drying at room temperature, the samples were analyzed on a Bruker Autoflex III MALDI TOF-TOF Mass Spectrometer. MALDI-MS and MS/MS data were acquired and combined through the BioTools 3.0 program to search protein database (SwissProt 57.1, 462764 sequences; 163773385 residues) by using the in-house Mascot software (Matrix Science, London, UK). 

### 3.8. Statistical Analysis

The 2D-gel were scanned and the obtained 2D images were analyzed using Progenesis SameSpots (Nonlinear Dynamics, Newcastle upon Tyne, UK) software. The images went through a process of image quality assessment first. Any positional errors introduced during scanning can be corrected using in-built tools (flip, rotate, invert) before proceeding with the analysis. By using the automated image alignment function, the images can be well aligned at the pixel level, making it possible to accurately compare images by removing the positional variation introduced during the electrophoresis process, thus enabled well matching of gels. The up- and down-regulated spots were identified by comparing the relative abundance of each matched protein spot between model control group and TGS-treated group (*n* = 3 for each group, gels were run in duplicate for each sample). The expression level of differentially expressed proteins more than 1.5 folds between two groups with an ANOVA *p*-value lower than 0.05 were considered to be significant.

## 4. Conclusions

In the current study we showed the global protein image profiles of the left ventricle of isolated rat heart perfused in Langendorff system under ischemia-reperfusion injury condition with or without TGS treatment for the first time. Based on the identification of differentially expressed proteins in TGS-treated hearts, it could be concluded that improvement of cardiac energy metabolism through modulation of TCA in the isolated rat hearts in Langendorff system is probably one of the major molecular mechanisms of the action of TGSs against heart injury caused by ischemia-reperfusion. This newly-revealed mechanism has provided evidence for better understanding of biochemical responses of Radix Ginseng which has been used with a long history, and also sheds light on a possible therapeutic strategy for ischemic heart diseases. 
